# Bioinformatics Methods and Biological Interpretation for Next-Generation Sequencing Data

**DOI:** 10.1155/2015/690873

**Published:** 2015-09-07

**Authors:** Guohua Wang, Yunlong Liu, Dongxiao Zhu, Gunnar W. Klau, Weixing Feng

**Affiliations:** ^1^School of Computer Science and Technology, Harbin Institute of Technology, Harbin, Heilongjiang 150001, China; ^2^Center for Computational Biology and Bioinformatics, Department of Medical and Molecular Genetics, Indiana University School of Medicine, Indianapolis, IN 46202, USA; ^3^Department of Computer Science, Wayne State University, Detroit, MI 48202, USA; ^4^Life Sciences, Centrum Wiskunde & Informatica, Science Park 123, 1098 XG Amsterdam, Netherlands; ^5^Pattern Recognition and Intelligent System Institute, Automation College, Harbin Engineering University, Harbin, Heilongjiang 150001, China

Next-generation sequencing (NGS) technologies have revolutionarily reshaped the landscape of “-omics” research areas and their effects are becoming increasingly widespread. With its significantly lower costs and higher throughput, NGS has been applied to genome, transcriptome, and epigenome research. The plethora of information that emerges from large-scale next-generation sequencing experiments has triggered the development of bioinformatics tools and method for efficient analysis, interpretation, and visualization of NGS data. Such methods and tools will substantially promote the life-science community to better and efficiently help understand the underlying biological principles and mechanisms. This special issue mainly focuses on the original research articles as well as review articles that develop new bioinformatics approaches, present novel platforms and systems, and describe concise models well explaining the biological context and application in relation to genetics, metagenomics, and clinical study from NGS data.

This special issue contains nine papers. Two papers discuss the application of NGS data analysis in metagenomics and one paper presents R package for metagenomic systems biology analysis. One review paper discusses the software to detect alternative splicing isoforms from deep sequencing data. The other five papers are related to application of NGS data integration in genomics, genetics, and epigenetics.

In “mmnet: An R Package for Metagenomics Systems Biology Analysis,” the authors developed R package, mmnet, to implement community-level metabolic network reconstruction and also implement a set of functions for automatic analysis pipeline construction. The package has substantial potentials in metagenomic studies that focus on identifying system-level variations of human microbiome associated with disease.

The paper “Constructing a Genome-Wide LD Map of Wild* A. gambiae* Using Next-Generation Sequencing” sequenced the genomes of nine individual wild* A. gambiae* mosquitoes using next-generation sequencing technologies. And 2,219,815 common single nucleotide polymorphisms (SNPs) were detected. Nearly one million SNPs that were genotyped with 99.6% confidence were extracted from these high-throughput sequencing data. Based on these SNP genotypes, the authors constructed a genome-wide linkage disequilibrium (LD) map for wild* A. gambiae* mosquitoes from malaria-endemic areas in Kenya and made it available through a public website.

The paper entitled “How to Isolate a Plant's Hypomethylome in One Shot” provided an easy, fast, and cost-effective tool to obtain a plant's hypomethylome (the nonmethylated part of the genome) by an optimized methyl filtration protocol with subsequent next-generation sequencing, in essence a variant of MRE-seq. The hypomethylomes which were identified in three plant species,* Oryza sativa*,* Picea abies*, and* Crocus sativus*, showed clear enrichment in genes and their flanking regions. This method is extremely conducive to studying and understanding the genomes of nonmodel organisms.

In “Genetic Interactions Explain Variance in Cingulate Amyloid Burden: An AV-45 PET Genome-Wide Association and Interaction Study in the ADNI Cohort,” the authors performed a genome-wide association study (GWAS) and a genome-wide interaction study (GWIS) of an amyloid imaging phenotype, using the data from Alzheimer's Disease (AD) Neuroimaging Initiative. The GWAS analysis revealed significant hits within or proximal to APOE, APOC1, and TOMM40 genes. The GWIS analysis yielded 8 novel SNP-SNP interaction findings that warrant replication and further investigation.

The paper “Understanding Transcription Factor Regulation by Integrating Gene Expression and DNase I Hypersensitive Sites” identified the functional transcription factor binding sites in gene regulatory region by integrating the DNase I hypersensitive sites with known position weight matrices. The authors present a model-based computational approach to predict a set of transcription factors that potentially cause such differential gene expression in cervical cancer HeLa S3 cell and HelaS3-ifna4h cell. This model demonstrated the potential to computationally identify the functional transcription factors in gene regulation.

The paper “Survey of Programs Used to Detect Alternative Splicing Isoforms from Deep Sequencing Data In Silico” is a review paper. Alternative splicing (AS) is very important for gene expression and protein diversity. First the authors summarized the alternative splicing forms and the means of selective splicing. Then the authors described the numerous methods for the read mapping of RNA-seq data and alternative types of splicing prediction software. At last, HMMSplicer, SOAPsplice, TopHat, and STAR were used to evaluate the performance of alternative splicing isoforms detection.

The article “MicroRNA Promoter Identification in* Arabidopsis* Using Multiple Histone Marker” was devoted to a computational strategy, which identified the promoter regions of most microRNA genes in* Arabidopsis*, using the genome wide profiles of nine histone markers. Based upon the assumption that the distributions of histone markers around the transcription start sites (TSSs) of microRNA genes are similar with the TSSs of protein coding gene, the Support Vector Machine (SVM) was used to identify 42 independent miRNA TSSs and 132 miRNA TSSs which are located in the promoters of upstream genes. The annotation of microRNA TSSs will provide the measurements regarding the initiation of transcription and better understanding of microRNA regulation.

The paper “454-Pyrosequencing Analysis of Bacterial Communities from Autotrophic Nitrogen Removal Bioreactors Utilizing Universal Primers: Effect of Annealing Temperature” carried out a metagenomic analysis (pyrosequencing) of total bacterial diversity including Anammox population in five autotrophic nitrogen removal technologies, two bench-scale (MBR and low temperature CANON) and three full-scale (Anammox, CANON, and DEMON), by optimization of primer selection and PCR conditions. The pyrosequencing data showed that annealing temperature of 45°C yielded the best results in terms of species richness and diversity for all bioreactors analyzed.

The paper entitled “Active Microbial Communities Inhabit Sulphate-Methane Interphase in Deep Bedrock Fracture Fluids in Olkiluoto, Finland” investigated active microbial communities of deep crystalline bedrock fracture water from seven different boreholes in Olkiluoto (Western Finland), using bacterial and archaeal 16S rRNA,* dsr*B, and* mcr*A gene transcript targeted 454 pyrosequencing. The results demonstrated that active and highly diverse but sparse and stratified microbial communities inhabited the Fennoscandian deep bedrock ecosystems.

